# Early-life adversity and cortisol response to social stress: a meta-analysis

**DOI:** 10.1038/s41398-017-0032-3

**Published:** 2017-12-11

**Authors:** Ioana Maria Bunea, Aurora Szentágotai-Tătar, Andrei C. Miu

**Affiliations:** 1 Cognitive Neuroscience Laboratory, Department of Psychology, Babeș-Bolyai University, Cluj-Napoca, 400084 Romania; 2 Department of Clinical Psychology and Psychotherapy, Babeș-Bolyai University, Cluj-Napoca, 400084 Romania

## Abstract

Early-life adversity has been associated with a life-long increased risk for psychopathology and chronic health problems. These long-term negative effects have been explained through stress sensitization, which may involve dysregulation of the hypothalamic–pituitary–adrenal (HPA) axis through either increased or decreased reactivity. The present meta-analysis assessed for the first time the effect of early-life adversity on cortisol response to social stress. Thirty data sets were included in the meta-analysis, in which early-life adversity and salivary cortisol response to social stress were assessed in 4292 individuals of different ages. Results indicated a moderate effect size (*g* = −0.39) in overall cortisol levels across studies. Separate analyses of cortisol at different stages of response showed large effect sizes at peak and recovery, and a moderate effect at baseline. Heterogeneity was large in this sample of studies and several moderators were identified. The effect size was larger in studies that focused on maltreatment compared to those that included other adversities, and in adults compared to children and adolescents. Percent of women in each sample and methodological quality were positive predictors of the effect size. Publication bias may be present, but the analysis was hampered by the high heterogeneity. Therefore, these results support the association between early-life adversity and blunted cortisol response to social stress, and they suggest that the long-term negative effects of early-life adversity may reach maximum levels in adults.

## Introduction

Childhood adversity, including maltreatment (i.e., abuse and neglect) and other traumatic events, has been compellingly associated with a life-long increased risk for psychopathology and chronic health problems^1–3^. For instance, studies in the National Comorbidity Survey Replication sample indicated that childhood adversity is associated with an increased risk for all common mental disorders^4^, which is maintained throughout the life course, as well as with higher functional impairment^5^ and persistence of these disorders^6^. Childhood adversity has also been related to poor health in adulthood and increased risk for coronary heart disease, hypertension, arthritis, diabetes, and migraine^7^. Overall, childhood adversity is linked to more years lost to disability than mental disorders^8^.

The long-term negative effects of childhood adversity have been explained through stress sensitization or an increased vulnerability to subsequent stressful events^9, 10^. This vulnerability may involve persistent dysregulation of stress responses, which is thought to result from exposure to stressors during sensitive developmental periods, when physiological systems undergo maturational changes^11, 12^. One of the stress response systems that has been extensively examined as a potential mechanism underlying stress sensitization is the hypothalamic–pituitary–adrenal (HPA) axis. The HPA axis undergoes protracted development throughout childhood and adolescence and may be particularly sensitive to early-life adversity^13^. In addition, cortisol, which is the main hormonal output of this system and is essential for meeting the increased metabolic demands of responding to stress, can also contribute, when dysregulated, to allostatic load and health deterioration^14, 15^. Cortisol dysregulation can involve both increased and decreased responses^12,14,15^, and an influential hypothesis has argued that blunted cortisol levels may develop as an adaptation of the HPA axis to sustained periods of hyper-reactivity^16, 17^.

Studies in the last two decades have repeatedly associated childhood adversity with blunted activity of the HPA axis, assessed using circadian or acute stress levels of cortisol^12,17^. However, a recent meta-analysis failed to replicate previous reports of blunted circadian cortisol slope and cortisol-awakening response in individuals with a history of childhood maltreatment^18^. Nonetheless, it remains possible that childhood adversity is associated with blunted cortisol response to mental stress. In contrast to circadian cortisol, which is specifically regulated by the suprachiasmatic nucleus^19^, the HPA response to psychological stress depends on a more extensive neural circuit that includes structures such as the hippocampus, the amygdala, and the prefrontal cortex, which are known to be sensitive to childhood adversity^12^. To our knowledge, no meta-analysis until now has directly scrutinized the evidence suggesting HPA stress sensitization.

The present meta-analysis investigated the effect of early-life adversity on cortisol response to social stress. Threat of social evaluation is an effective trigger of HPA responses^20^ and, thus, social stress may offer an excellent model for studying cortisol stress sensitization. In addition to estimating the effect across studies, the present research also examined whether the magnitude of the effect differs between types of adversity, as well as between children, adolescents, and adults. Moreover, given previous evidence from studies with the Trier Social Stress Test (TSST)^21^, we investigated sex differences and several elements of methodological quality as potential moderators.

## Materials and methods

### Identification of studies

Articles were identified by searching the PubMed, PsycINFO, and Embase electronic databases, from their inception to March 2017, crossing keywords, including truncated terms, related to early-life adversity (i.e., childhood or early life and trauma or adverse or stress or maltreatment) with those related to stress reactivity (i.e., social or acute stress) and HPA axis (i.e., cortisol). We also checked the reference list of eligible articles and other meta-analyses.

Studies were included if they: (1) involved human participants of all ages; (2) investigated early-life adversity, including maltreatment and other major stressful events; (3) induced social stress through threat of task performance evaluation by other people; and (4) assessed salivary cortisol. Therefore, we focused on forms of adversity that are typically traumatic and chronic^4^, and occurred during childhood and adolescence. Moreover, we included studies in which acute stress was characterized by social-evaluative threat and uncontrollability, in light of previous evidence that these elements are potent elicitors of cortisol responses^20^. Finally, considering that the majority of studies focused on salivary cortisol and those which assessed plasma cortisol^22, 23^ reported total rather than free cortisol, we limited the present meta-analysis to the former category. Exclusion criteria were related to: (1) the absence of control participants with below cutoff or no early-life adversity; (2) a focus on contextual variables (e.g., low income) that are not necessarily linked to adversity, or on recent stress, without controlling for early-life adversity; (3) exposure to prenatal stress (e.g., maternal substance abuse or exposure to violence); (4) substance (e.g., cocaine) abuse; and (5) the presence of a medical condition (e.g., diabetes, chronic fatigue). The relevance of every article was first assessed based on the title and abstract and then the full text. Two assessors independently examined full texts and selected eligible studies.

### Data extraction and risk of bias

Data from each study were extracted by one of the authors and checked by a second author. If the data reported in the article were insufficient to compute effect sizes, the authors were contacted and, eventually, the study was excluded if the data were not provided.

Risk of bias (RoB) was independently evaluated by two authors within eight domains that were selected a priori. RoB was evaluated as low if a study reported: (1) a direct assessment of early-life adversity (vs., e.g., history of institutionalization and adoption); (2) an assessment of the severity of early-life adversity; (3) no other task during saliva-sampling in addition to the social stress test; (4) social stress test scheduled in the afternoon (i.e., later than 12 p.m.); (5) at least three saliva samples to capture cortisol change; (6) a pre-stress assessment of salivary cortisol; (7) an assessment of cortisol in the first 30 min. following stress onset, to capture peak cortisol; and (8) a manipulation check documenting the expected cortisol rise from pre-stress to post-stress. Inter-rater agreement was examined and disagreements were resolved by discussion.

### Statistical analysis

All analyses were run using the Comprehensive Meta-Analysis version 2.0 software (Biostat, Englewood, NJ, USA). Where available, means and standard deviations of salivary cortisol were extracted from text and tables, as well as from graphs using the Web Plot Digitizer software^24^. Alternatively, effect sizes were estimated based on correlations or unstandardized regression coefficients and sample sizes, as well as results from statistical tests (e.g., ANOVA) of differences between groups with a known number of participants, using formulae implemented in comprehensive meta-analysis^25^.

For the main analysis, data across multiple assessments were averaged. Effect sizes were estimated based on Hedge’s *g* coefficient, corrected for small sample sizes^26^, and were pooled using a random-effects model. We interpreted effect sizes smaller than 0.32 as small, 0.33–0.55 as moderate, and higher than 0.56 as high^27^. Heterogeneity was assessed using *I* ^2^ statistic, with values of 25%, 50%, and 75% defined as low, moderate, and high levels, respectively.

A follow-up analysis estimated the effect size at baseline, peak, and recovery assessments in a subset of studies from which these data were available. Following Liu et al.^28^, the peak was defined as the highest cortisol value in the first 30 min. following stress onset, and recovery was estimated based on the cortisol level at the assessment that was closest to 60 min. following stress onset.

Subgroup analyses based on a mixed-effect model were used to investigate categorical moderators, which included the type of early-life adversity (i.e., maltreatment vs. other adversities), the developmental stage at the time of stress assessment (i.e., children vs. adolescents vs. adults), and the method used to assess early-life adversity (i.e., questionnaires vs. interviews vs. official records)^25^. Throughout the meta-analysis, we restricted moderator analyses to cases where each group included at least five studies. Two continuous moderators, namely the percent of female participants in each study and an overall RoB score (i.e., with one point earned for each low-risk rating), were investigated using meta-regression^25^. Publication bias was examined using inspection of the funnel plot, Egger’s test for funnel plot asymmetry^25^, and the trim-and-fill procedure^29^.

## Results

### Study selection

**Fig. 1:**
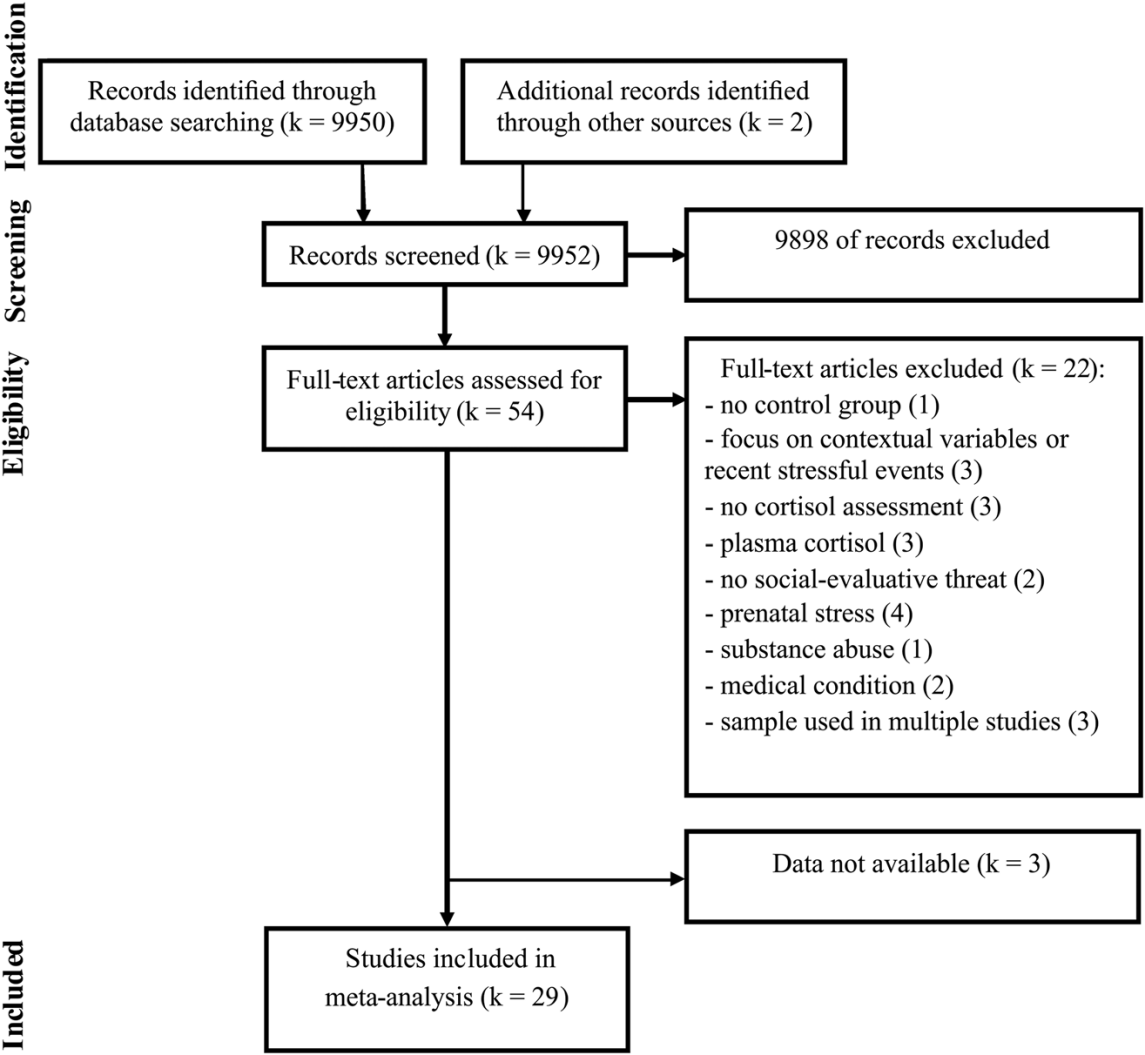
Flow diagram describing the process of study selection

As shown in the flow diagram (Fig. 1), the systematic search returned 9952 citations. After screening the titles and abstracts, 9898 studies were discarded because they were duplicates or did not meet the inclusion criteria. The full text of the remaining 54 articles was examined in more detail, and we found that another 22 studies did not meet the criteria. The remaining 32 studies met the inclusion criteria and 24 of them had enough data for calculating the effect sizes. The authors of the eight studies from which we needed additional information were contacted and data were obtained from five of these studies. Data could not be obtained from the authors of the remaining three studies^30–32^, which were consequently not included in the meta-analysis.

### Study characteristics

The 29 articles^33–61^ included in the meta-analysis contained 30 data sets, which involved 4292 participants, with ages ranging from 8 to 62 years (Supplementary Table 1). All participants were healthy volunteers. Four studies included clinical groups (i.e., patients with major depressive disorder), but, given that they were too few for a moderation analysis, we only extracted the data from the control groups with or without early-life adversity.

All studies investigated the effect of early-life adversity on salivary cortisol response to stress. Studies focused on early-life maltreatment (i.e., physical and emotional abuse and neglect, sexual abuse; *k* = 21), other major adversities (i.e., parental conflict and witnessing violence; separation from parents and institutionalization; parental mental illness and substance abuse; illness; death of family; loneliness and relational problems; and financial problems; *k *= 2), or a combination of these (*k* = 7). All adversities were reported as having occurred before age 18, “while growing up”, or until the present in children and adolescents (Supplementary Table 1). Childhood adversity was assessed using self-reported questionnaires in the majority of studies (*k* = 16), but also through interviews (*k* = 6), official records of childhood maltreatment (*k* = 4), and history of institutionalization and adoption (*k* = 4; Supplementary Table 1). The majority of studies (*k* = 22) compared cortisol levels between groups of participants with different histories of childhood adversity. The rest of the studies (*k* = 8) described the relations between continuous measures of childhood adversity and cortisol responses (Supplementary Table 1).

In most studies, stress was induced through the TSST for adults or children (*k* = 24), as well as similar protocols that also involved public speaking and a cognitive task (i.e., Groninger Social Stress Test; Psychosocial Stress Test; *k* = 2). The rest of the studies (*k* = 4) used verbal interaction (e.g., Noisy Neighbor task), and cognitive (e.g., mental arithmetic, including the Montreal Imaging Stress Task; Stroop task) or motor tasks (e.g., mirror tracing; Supplementary Table 1). All the stress induction protocols involved social-evaluative stress induced through the presence of an evaluative audience during the task, social comparison, and/or video recording of the performance for subsequent evaluation. Salivary cortisol was assessed in all the studies, but the number of assessments ranged between 2 and 12.

RoB assessments using the eight a priori criteria (Supplementary Table 2) showed high inter-rater agreement (Cohen’s *k* > 0.90). Four studies did not report a direct assessment of childhood adversity and instead relied on history of institutionalization and adoption. In eight studies, severity was not considered in the assessment of adverse events. In four studies, the social stress test was combined with other tasks during the saliva-sampling interval. Some studies were partially scheduled in the morning (*k* = 3) or the time of day of the stress test was not reported (*k* = 2). One study relied on less than three cortisol assessments. All studies assessed salivary cortisol pre-stress and during the first 30 min post-stress. The cortisol increase following stress was not significant in six studies, and this manipulation check was not reported in another three studies. Overall, 10 studies were rated as low RoB on all eight criteria.

### Main effects

**Fig. 2:**
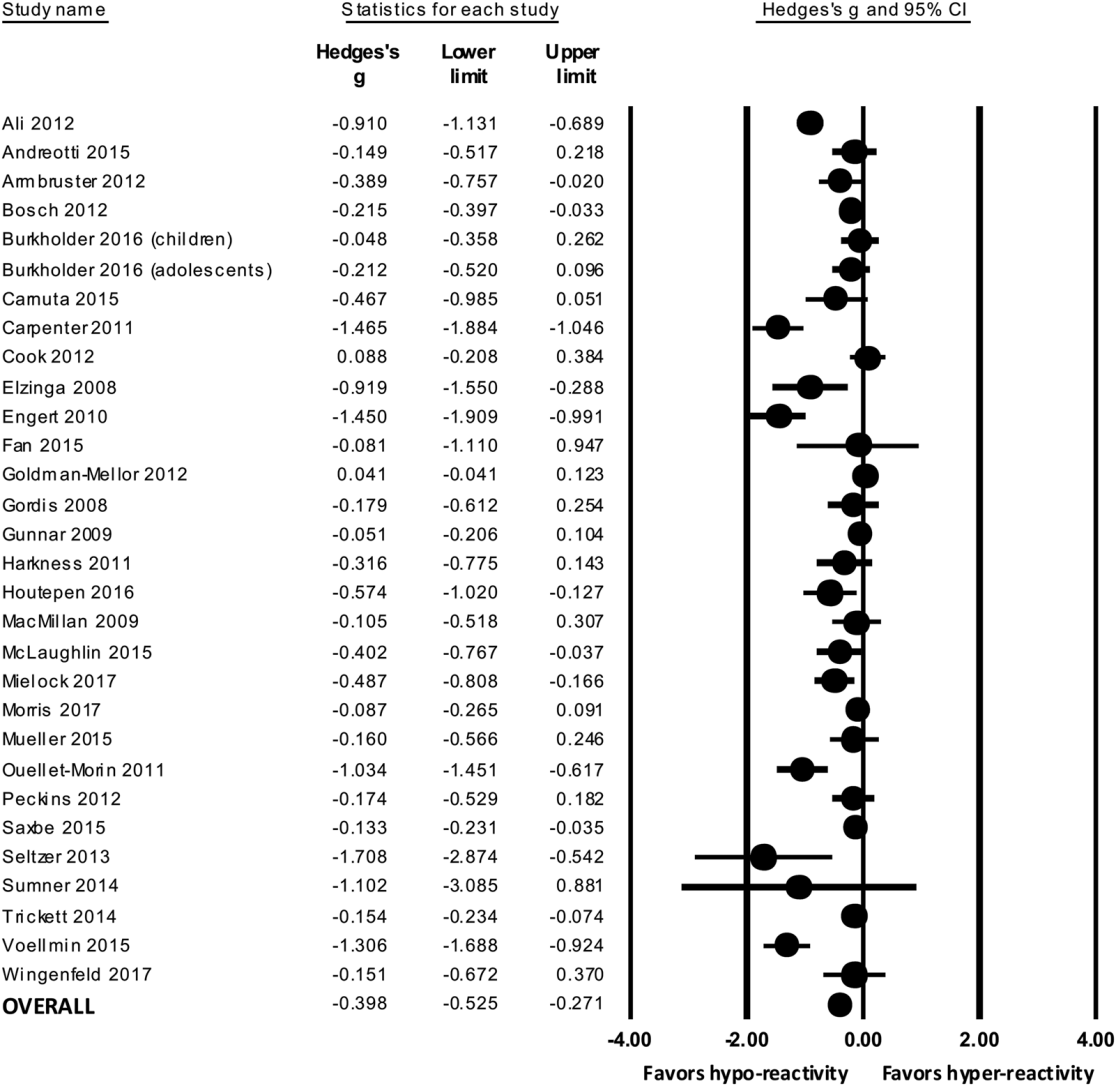
Forest plot showing the individual and pooled effect sizes of early-life adversity and cortisol response to social stress

Fig. 2 shows the individual effect sizes from all studies included in the pooled analysis. The average effect size across studies was −0.39 (95% CI: −0.52, −0.27), with lower cortisol responses in participants with childhood adversity. Heterogeneity was high (*I* ^2^ = 86.63%; *p* < 0.001), which suggests that other factors may interact with early-life adversity in modulating cortisol responses to social stress.

We analyzed the overall effect size at baseline (*k* = 18), peak (*k* = 18), and recovery (*k* = 15) assessments of cortisol. Supplementary Table 3 shows levels of cortisol at all three assessments in each individual study. Early-life adversity was associated with blunted cortisol at all three assessments (all *p* values < 0.01), with a moderate effect size at baseline (Hedge’s *g* = −0.33; 95% CI: −0.58, −0.08) and large effect sizes at peak (Hedge’s *g* = −0.77; 95% CI: −1.09, −0.44) and recovery (Hedge’s *g* = −0.77; 95% CI: −1.14, −0.39).

### Subgroup and meta-regression analyses

The effect size of blunted cortisol reactivity was larger in studies that focused on maltreatment (*k* = 22; Hedge’s *g* = −0.49; 95% CI: −0.65, −0.32) compared to studies that included other forms of adversity (*k* = 8; Hedge’s *g* = −0.14; 95% CI: −0.24, −0.04; *p* for interaction < 0.001). In addition, the effect size indicating blunted cortisol responses to stress was larger in adults (*k* = 13; Hedge’s *g* = −0.63; 95% CI: –0.97, –0.28) compared to children (*k* = 9; Hedge’s *g* = −0.25; 95% CI: −0.38, −0.11) and adolescents (*k* = 8; Hedge’s *g* = −0.13; 95% CI: −0.23, −0.03; *p* for interaction = 0.020). The effect size was also larger in studies in which early-life adversity was assessed using self-reported questionnaires (*k* = 12; Hedge’s *g* = −0.61; 95% CI: −0.98, −0.25) compared to interviews (*k* = 9; Hedge’s *g* = −0.33; 95% CI: −0.52, −0.15) and official records (*k* = 9; Hedge’s *g* = −0.14; 95% CI: −0.22, −0.06; *p* for interaction = 0.011).

Meta-regression indicated that both the percent of female participants (slope *β* = −0.01; 95% CI: −0.02, −0.01; *p* = 0.006) and the number of low RoB ratings (slope *β* = −0.13; 95% CI: −0.24, −0.03; *p* = 0.007) were significant positive predictors of the effect size of blunted cortisol reactivity (see Supplementary Figs. 1 and 2). Follow-up subgroup analyses could be run on three separate RoB domains, which included at least five studies in each category. These analyses indicated that the effect size was higher in low-risk studies, which reported significant pre-stress to post-stress changes in cortisol (*p* for interaction = 0.004), compared to those in which this change was not significant. The effect size difference was marginally significant between studies, which considered or not the severity of adversity (*p* for interaction = 0.097), with a larger effect size in the former category. The effect size was similar in studies run at partially different times of day.

### Publication bias

Inspection of the funnel plot (Supplementary Fig. 3) and Egger’s test (intercept *B* = −2.40; 95% CI: −3.77, −1.03; *p* = 0.001) suggested publication bias. The studies included in the meta-analysis were evenly nested within both sides of the funnel, but clustered toward the peak, which suggests that the publication of large studies may have been favored. However, the trim-and-fill procedure did not support publication bias, with identical point estimates of the overall effect size for observed and adjusted values.

## Discussion

The present meta-analysis included 30 data sets, in which early-life adversity and salivary cortisol response to social stress were assessed in 4292 individuals of different ages. In line with the HPA stress sensitization hypothesis and evidence suggesting that reduced activity is a form of HPA dysregulation, the present results support the view that cortisol response to social stress is blunted in individuals with a history of early-life adversity.

There was a moderate effect suggesting lower levels of cortisol overall in association with early-life adversity. However, the difference suggesting blunted cortisol in individuals with early-life adversity was large at the peak and recovery phases of the stress response, and moderate at baseline. This suggests that the effects of early-life adversity on cortisol may be more apparent during the acute phases of the stress response. This may explain why a previous meta-analysis^18^, which focused on resting cortisol measures (i.e., circadian slope and cortisol-awakening response) failed to find an effect of childhood maltreatment. Having focused on reactivity to social stressors, which are potent inducers of HPA responses due to their elements of social-evaluative threat and uncontrollability^20^, this meta-analysis does not address the possibility that cortisol response to other types of stress (e.g., pain) may also be altered following early-life adversity.

Several significant moderators were identified and could explain the large heterogeneity found in this sample of studies. The overall effect size was moderate across the studies that focused on maltreatment and small in those which included other early-life adversities. This is in line with previous results showing that compared to other childhood adversities (e.g., parental separation, physical illness), maltreatment and other forms of maladaptive family functioning are associated with higher risk^4^ and persistence^6^ of psychopathology.

The present results also indicate that the effect of early-life adversity on cortisol response to social stress is large in adults and small in children and adolescents. A previous longitudinal study^16^ has shown that cortisol becomes blunted following a period of HPA hyperactivity and reaches maximum levels in adults with a history of childhood maltreatment. Therefore, the small effect size of blunted cortisol response in children and adolescents may reflect an early phase of HPA dysregulation. We also found that the effect size of blunted cortisol response was larger in studies that used questionnaires compared to interviews and official records. This is surprising, given that early-life adversity questionnaires may be prone to under-reporting^62^, which can contribute to an underestimation of effects. Therefore, considering that the majority (83%) of questionnaire studies were in adults, it is possible that the larger effect size found in these studies is driven by developmental rather than methodological differences. However, the present meta-analysis cannot disentangle these variables because there are too few studies to stratify comparisons between methods of assessment by age group.

Meta-regression results also suggested that cortisol reactivity to social stress was larger in studies that included more women. This is in line with a recent meta-analysis^28^, which found reduced cortisol responses to social stress in women compared to men, specifically at peak and recovery phases of stress. There is also evidence that lower levels of recent stress are necessary to trigger stress sensitization in liability to post-traumatic stress disorder in women with a history of childhood adversity, compared to men^10^. Therefore, the present association between the percent of women in the sample and the effect size of blunted cortisol response in individuals with early-life adversity may reflect either sex differences in HPA reactivity, or enhanced stress sensitization effects, or both. There was a also trend for a larger effect size in studies that included an assessment of the severity of early-life adversity. While one may indeed expect a dose–response relation between severity of adversity and blunted cortisol, the modest difference uncovered in the present study may be explained by our focus on adverse events that are typically traumatic and chronic^4^.

The present meta-analysis is limited by several factors. First, there may be publication bias in the present sample of studies, although the large heterogeneity could have conflated statistical estimates of funnel asymmetry^63^ and thus precludes us from drawing a firm conclusion on this issue. Second, only a third of studies met all methodological requirements, which suggests that RoB is present in many studies. Meta-regression results suggested that high RoB studies may underestimate the blunted cortisol response in individuals with early-life adversity. Third, the low number of studies did not allow us to investigate combinations of moderators, such as age category and assessment method. Finally, only four studies included groups with psychopathology (i.e., major depression) and, therefore, examination of clinical status as moderator was not possible. Future studies comparing clinical and nonclinical samples are warranted, given that blunted cortisol reactivity has been consistently found in major depression and anxiety disorders^64^.

In conclusion, this meta-analysis supports the association between early-life adversity, particularly childhood maltreatment, and blunted cortisol response to social stress. It provides the first meta-analytic evidence for the long-term effect of early-life adversity on HPA axis reactivity to social stress, and suggests that this effect reaches maximum levels in adults. These results could have clinical implications considering that HPA stress sensitization may be one of the mechanisms that could explain the life-long vulnerability to psychopathology that develops following early-life adversity.

## Electronic supplementary material

Supplementary Information
