# Supplementary material for: Early-life adversity and cortisol response to social stress: a meta-analysis
Source: Transl Psychiatry. 2017 Dec 11;7:1274. doi: 10.1038/s41398-017-0032-3 (PMC5802499; doi:10.1038/s41398-017-0032-3)
Supplement: Supplementary file 1 — Supplementary Information [file 41398_2017_32_MOESM1_ESM.docx]

**Supplementary Information**

Supplementary Figs. 1-3

Supplementary Tables 1-3

**Supplementary Fig. 1** Effect size of cortisol blunting associated with early adversity regressed on percent of female participants.

**Supplementary Fig. 2** Effect size of cortisol blunting associated with early adversity regressed on the number of low risk ratings of studies included in the meta-analysis.

**Supplementary Fig. 3** Funnel plot showing the distribution of effect sizes as a function of study size.

**Supplementary Table 1**

The characteristics of studies included in the meta-analysis

| Study | N | Mean age (range) | Women (%) | Childhood adversity | Method of assessment | Method of stress induction | Time of day | Saliva sampling relative to stress onset | Outcome | Measures of psychopathology |
| --- | --- | --- | --- | --- | --- | --- | --- | --- | --- | --- |
| Ali (2012)[^1^](#_ENREF_1) | 37 | 25.75 (18-35) | 51 | Care and overprotection from mother and father; physical abuse and neglect; emotional abuse and neglect; sexual abuse | Childhood Trauma Questionnaire; Parental Bonding Instrument | TSST | Afternoon | -10; 0; +10; +20; +30; +40; +50; +60; +70 | Difference (low care vs. high care) | NR |
| Andreotti (2015)[^2^](#_ENREF_2) | 116 | 18.96 (18-22) | 100 | Conflict in the home | Children Perception of Inter-parental Conflict Scale | NNT | Afternoon | One pre-stress and 4 post-stress samples (task duration not reported) | Correlation | NR |
| Armbruster (2012)[^3^](#_ENREF_3) | 119 | 9.32 (8-12) | 43.6 | Illness; family problems; problems related to schooling; negative socio-economic circumstances; social problems with peers; disaster; death of significant others | Life History Calendar interview | TSST-C | Afternoon | -2; +2; +10; +20; +30 | Correlation | NR |
| Bosch (2012)[^4^](#_ENREF_4) | 471 | 14-18 | 53 | Loneliness; physical abuse; sexual abuse; bullying; lack of friends; conflicts; severe problems of family members or friends; out of home placement; running away from home | Semi-structured interview related to number and duration of adversities | GSST | Morning and afternoon | 0; +24; +44; +64 | Difference (low vs. intermediate vs. high amount of chronic stress) | NR |
| Burkholder (2016) (children)[^5^](#_ENREF_5) | 79 | 9.83 | 50 | Institutionalization | NR | TSST-C | Afternoon | 0; +20 | Difference (adopted vs. never adopted) | NR |
| Burkholder (2016) (adolescents)[^5^](#_ENREF_5) | 82 | 15.81 | 51 | Institutionalization | NR | TSST-C | Afternoon | 0; +20 | Difference (adopted vs. never adopted) | NR |
| Carnuta (2015)[^6^](#_ENREF_6) | 62 | 20.85 | 100 | Physical abuse; sexual abuse; major parental conflicts, divorce or separation; death of a family or close friend; severe illness or injury | Childhood Traumatic Events Scale | TSST | Afternoon | 0; +5; +15; +20; +30 | Correlation | NR |
| Carpenter (2011)[^7^](#_ENREF_7) | 110 | 32.8 (18-61) | 100 | Physical abuse and neglect; emotional abuse and neglect; sexual abuse | Childhood Trauma Questionnaire | TSST | Afternoon | 0; +15; +30; +45; +60; +75; +90 | Difference (physical abuse vs. no physical abuse) | NR |
| Cook (2012)[^8^](#_ENREF_8) | 175 | 15.36 | 51.8 | Physical abuse and neglect; emotional abuse and neglect; sexual abuse | Childhood Trauma Questionnaire | TSST-C | Afternoon | 0; +15; +30; +45; +60; +75 | Difference (low vs. high child maltreatment) | NR |
| Elzinga (2008)[^9^](#_ENREF_9) | 80 | 21.6 | 65 | Emotional abuse and neglect; physical abuse, sexual abuse, sexual harassment, general traumatic events | Traumatic Experiences Checklist | TSST | Morning and afternoon | -20; -1; +15; +40; +55 | Difference (low vs. high adverse events) | NR |
| Engert (2010)[^10^](#_ENREF_10) | 34 | 21.08 (18-30) | 30 | Care and overprotection from mother and father | Parental Bonding Instrument | TSST | Afternoon | -20; -10; 0; +10; +20; +30; +40; +50; +60 | Difference (low vs. medium care) | NR |
| Fan (2015)[^11^](#_ENREF_11) | 31 | 28.3 (21-37) | 0 | Physical abuse and neglect; emotional abuse and neglect; sexual abuse | Childhood Trauma Questionnaire | MIST | NR | 0; +21 | Correlation | NR |
| Goldman-Mellor (2012)[^12^](#_ENREF_12) | 543 | 62.7 | 39 | Physical abuse; separation from mother or time spent in orphanage for >1 years; parental death; serious familial mental illness or substance abuse; parental divorce; frequent parental conflict; harsh parenting style | Heart Scan Study or Whitehall Wave 5 Study Questionnaires | Stroop task; mirror tracing task | Morning and afternoon | 0; +10; +30; +55; +85 | Difference (no early life adversity vs. early life adversity without distress vs. early life adversity with minimal or recurrent distress | Psychological distress assessed with the General Health Questionnaire |
| Gordis (2008)[^13^](#_ENREF_13) | 84 | 12.1 (9.1-14.5) | 47 | Physical abuse; sexual abuse; neglect | Official report | TSST-C | Afternoon | -45; -10; +15; +25; +35; 45 | Correlation | NR |
| Gunnar (2009)[^14^](#_ENREF_14) | 124 | 11.25 (10.02-12.21) | 48 | Institutionalization | NR | TSST-C | Afternoon | -30; -15; 0; +6; +16; +26; +36; +46 | Difference (late adopted vs. never adopted) | NR |
| Harkness (2010)[^15^](#_ENREF_15) | 71 | 15.39 (12-21) | 67 | Antipathy; indifference; physical abuse; sexual abuse | Childhood Experience of Care and Abuse | TSST | Afternoon | 0; +10; +30; +90; +150 | Difference (no maltreatment vs. maltreatment | MDD assessed using the Schedule of Affective Disorders and Schizophrenia |
| Houtepen (2016)[^16^](#_ENREF_16) | 85 | 32.5 | 49 | Physical abuse and neglect; emotional abuse and neglect; sexual abuse | Childhood Trauma Questionnaire | TSST (group) | Afternoon | One pre-stress and 7 post-stress samples (unknown time) | Correlation | NR |
| MacMillan (2009)[^17^](#_ENREF_17) | 67 | 14.09 | 100 | Physical abuse; sexual abuse; emotional abuse and neglect; neglect; witnessing domestic violence; physical assault; sexual assault | Childhood Experiences of Violence Questionnaire; Childhood Trauma Questionnaire | TSST-C | Afternoon | -45; -25; -5; +20; +40; +60 | Correlation | NR |
| McLaughlin (2015)[^18^](#_ENREF_18) | 138 | 12.90 | NA | Institutionalization | NR | TSST | NR | One pre-stress and 4 post-stress samples (unknown time) | Difference (care as usual vs. foster care vs. never institutionalized) | NR |
| Mielock (2017)[^19^](#_ENREF_19) | 52 | 27.45 | 100 | Separation/los; life-threatening illness or injury; physical neglect; emotional abuse; physical abuse or assault; witnessing domestic violence; sexual abuse or assault | Childhood Adversity Interview | TSST | Afternoon | -120; -90; -60; -30; -1; +10; +20; +30; +40; +50; +60; +70 | Difference (healthy vs. maltreated without MDD vs. maltreated with MDD) | MDD assessed using the Structured Clinical Interview for DSM-IV |
| Morris (2017)[^20^](#_ENREF_20) | 145 | 14.46 (12-17) | 47 | Physical abuse/ assault; sexual abuse/assault; witnessing domestic violence | Childhood Adversity Interview | TSST-C | Afternoon | -120; -90; -60; -30; -1, +10; +20; +30; +40; +50; +60; +70 | Difference (healthy vs. maltreated without MDD vs. maltreated with MDD) | MDD assessed using the Schedule for Affective Disorders and Schizophrenia for School-Age Children - Present and Lifetime Version |
| Mueller (2015)[^21^](#_ENREF_21) | 106 | 23.8 (19-31) | 47 | Death of close relatives or friends; serious illness or injury; relationship stressors; major difficulties at work or in school; financial problems; experienced disasters | Life History Calendar Interview | TSST | Afternoon | -2; +12; +22; +32; +42 | Difference (none vs. moderate vs. high stressful life events) | NR |
| Ouellet-Morin (2011)[^22^](#_ENREF_22) | 190 | 12 | 49.5 | Physical harm, bullying victimization | Interviews with mothers | PST | Afternoon | -20; -2; +15; +25; +35 | Difference (maltreated/bullied versus control) | NR |
| Peckins (2012)[^23^](#_ENREF_23) | 124 | 10.49 | 51 | Exposure to violence occurring within the past 12 months and during the child's lifetime | My Exposure to Violence | TSST-C | Afternoon | -20; -5; 0; +10; +20 | Correlation | NR |
| Saxbe (2015)[^24^](#_ENREF_24) | 277 | 10.84  (9-12) | 50 | Neglect; physical abuse; sexual abuse | Official report | TSST-C | Afternoon | -45; -10; +13; +23; +33; +43 | Difference (maltreated vs. control) | NR |
| Seltzer (2013)[^25^](#_ENREF_25) | 73 | 9  (8-11.5) | 53 | Physical abuse | Official report; Parent-Child Conflict Tactics Scale | TSST-C | Afternoon | 0; +16; +31; +46; +61; +76 | Differences(maltreated vs. control) | NR |
| Sumner (2014)[^26^](#_ENREF_26) | 168 | 14.9  (13-17) | 56 | Physical abuse and neglect; emotional abuse and neglect; sexual abuse | Childhood Trauma Questionnaire; Childhood Experience of Care and Abuse | TSST | Afternoon | -5; +15; +30 | Difference (no history vs. positive history of maltreatment) | NR |
| Trickett (2014)[^27^](#_ENREF_27) | 454 | 10.97 | 45 | Neglect; physical abuse; sexual abuse; emotional maltreatment | Official report | TSST-C | Afternoon | -30; -10; +14; +24; +34; +44 | Differences (abused vs. control) | NR |
| Voellmin (2015)[^28^](#_ENREF_28) | 104 | 21.7  (18-25) | 100 | General trauma; physical abuse; emotional abuse; sexual abuse | Early Trauma Inventory - Self Report | MIST | Afternoon | -5; 0; +22; +31; +46; +61; +76 | Difference (no adverse childhood experiences vs. adverse childhood experiences) | NR |
| Wingenfeld (2017)[^29^](#_ENREF_29) | 91 | 34.6 | 100 | Physical abuse; sexual abuse | Childhood Trauma Questionnaire | TSST | Afternoon | 0; +15; +25; +45; +60 | Difference (healthy vs. maltreated without MDD vs. maltreated with MDD) | MDD assessed with Structured Clinical Interview for DSM-IV axis I and II |

*Note.* Abbreviations: GSST, Groninger Social Stress Task; MDD, major depressive disorder; MIST, Montreal Imaging Stress Task; NNT, Noisy Neighbor Task; NR, not reported; PST, Psychosocial Stress Test ; TSST, Trier Social Stress Test; TSST-C, TSST for children.

**Supplementary Table 2**

Quality measures of studies included in the meta-analysis based on eight a priori markers of validity

| Study | Direct assessment of early adversity | Assessment of severity for childhood adversity^a^ | Separate induction without additional tasks | Stress test scheduled in the afternoon | More than three saliva samples | Pre-stress saliva sample | Post-stress saliva sample in the first 30 minutes after stress onset | Significant post-stress cortisol increase |
| --- | --- | --- | --- | --- | --- | --- | --- | --- |
| Ali (2012)[^1^](#_ENREF_1) | Yes | Yes | Yes | Yes | Yes | Yes | Yes | Yes |
| Andreotti (2015)[^2^](#_ENREF_2) | Yes | Yes | No | Yes | Yes | Yes | Yes | No |
| Armbruster (2012)[^3^](#_ENREF_3) | Yes | Yes | Yes | Yes | Yes | Yes | Yes | Yes |
| Bosch (2012)[^4^](#_ENREF_4) | Yes | Yes | No | No | Yes | Yes | Yes | Yes |
| Burkholder (2016) (children)[^5^](#_ENREF_5) | No | No | Yes | Yes | Yes^b^ | Yes | Yes | Yes^b^ |
| Burkholder (2016) (adolescents)[^5^](#_ENREF_5) | No | No | Yes | Yes | Yes^b^ | Yes | Yes | Yes^b^ |
| Carnuta (2015)[^6^](#_ENREF_6) | Yes | Yes | Yes | Yes | Yes | Yes | Yes | Yes |
| Carpenter (2011)[^7^](#_ENREF_7) | Yes | Yes | Yes | Yes | Yes | Yes | Yes | Yes |
| Cook (2012)[^8^](#_ENREF_8) | Yes | Yes | Yes | Yes | Yes | Yes | Yes | NR |
| Elzinga (2008)[^9^](#_ENREF_9) | Yes | Yes | Yes | No | Yes | Yes | Yes | Yes |
| Engert (2010)[^10^](#_ENREF_10) | Yes | Yes | Yes | Yes | Yes | Yes | Yes | Yes |
| Fan (2015)[^11^](#_ENREF_11) | Yes | Yes | Yes | NR | No | Yes | Yes | Yes |
| Goldman-Mellor (2012)[^12^](#_ENREF_12) | Yes | No | Yes | No | Yes | Yes | Yes | Yes |
| Gordis (2008)[^13^](#_ENREF_13) | Yes | Yes | Yes | Yes | Yes | Yes | Yes | No |
| Gunnar (2009)[^14^](#_ENREF_14) | No | No | Yes | Yes | Yes | Yes | Yes | Yes |
| Harkness (2010)[^15^](#_ENREF_15) | Yes | Yes | Yes | Yes | Yes | Yes | Yes | No |
| Houtepen (2015)[^16^](#_ENREF_16) | Yes | Yes | Yes | Yes | Yes | Yes | Yes | NR |
| MacMillan (2009)[^17^](#_ENREF_17) | Yes | Yes | Yes | Yes | Yes | Yes | Yes | Yes |
| McLaughlin (2015)[^18^](#_ENREF_18) | No | No | No | NR | Yes | Yes | Yes | Yes |
| Mielock (2017)[^19^](#_ENREF_19) | Yes | Yes | Yes | Yes | Yes | Yes | Yes | Yes |
| Morris (2017)[^20^](#_ENREF_20) | Yes | Yes | Yes | Yes | Yes | Yes | Yes | Yes |
| Mueller (2011)[^21^](#_ENREF_21) | Yes | No | Yes | Yes | Yes | Yes | Yes | Yes |
| Ouellet-Morin (2011)[^22^](#_ENREF_22) | Yes | No | Yes | Yes | Yes | Yes | Yes | Yes |
| Peckins (2012)[^23^](#_ENREF_23) | Yes | No | Yes | Yes | Yes | Yes | Yes | NR |
| Saxbe (2015)[^24^](#_ENREF_24) | Yes | Yes | Yes | Yes | Yes | Yes | Yes | No |
| Seltzer (2013)[^25^](#_ENREF_25) | Yes | Yes | Yes | Yes | Yes | Yes | Yes | No |
| Sumner (2014)[^26^](#_ENREF_26) | Yes | Yes | Yes | Yes | Yes | Yes | Yes | No |
| Trickett (2014)[^27^](#_ENREF_27) | Yes | Yes | Yes | Yes | Yes | Yes | Yes | Yes |
| Voellmin (2015)[^28^](#_ENREF_28) | Yes | Yes | Yes | Yes | Yes | Yes | Yes | Yes |
| Wingenfeld (2017)[^29^](#_ENREF_29) | Yes | Yes | No | Yes | Yes | Yes | Yes | Yes |

*Note.* Abbreviations: NR, not reported.

^a^ In studies in which maltreatment was documented based on history of child protection referrals, we assumed that severity was considered.

^b^ Four saliva samples were collected and they were reported in a previous study[^30^](#_ENREF_30).

**Supplementary Table 3**

Cortisol levels at baseline, peak and recovery in studies included in the follow-up meta-analysis

| Study | Time of peak cortisol post-stress, relative to stress onset | Time of recovery cortisol post-stress, relative to stress onset | Baseline cortisol (M ± SD, nmol/l) | | Peak cortisol (M ± SD, nmol/l) | | Recovery cortisol (M ± SD, nmol/l) | |
| --- | --- | --- | --- | --- | --- | --- | --- | --- |
|  |  |  | No early adversity | Early adversity | No early adversity | Early adversity | No early adversity | Early adversity |
| Ali (2012)[^1^](#_ENREF_1) | +30 | +70 | 3.80 ± 2.17 | 2.29 ± 0.91 | 7.90 ± 5.26 | 4.67 ± 3.42 | 4.74 ± 1.97 | 2.67 ± 1.30 |
| Burkholder (2016) (children)[^5^](#_ENREF_5) | +20 | NR | 3.31 ± 2.43 | 3.06 ± 2.24 | 3.73 ± 3.29 | 3.76 ± 3.41 | NR | NR |
| Burkholder (2016) (adolescents)[^5^](#_ENREF_5) | +20 | NR | 4.67 ± 2.37 | 4.43 ± 3.47 | 5.92 ± 3.82 | 4.62 ± 3.74 | NR | NR |
| Carpenter (2011)[^7^](#_ENREF_7) | +30 | +75 | 6.63 ± 1.21 | 4.33 ± 3.06 | 9.46 ± 1.69 | 3.09 ± 3.78 | 8.58 ± 2.27 | 5.06 ± 5.76 |
| Elzinga (2008)[^9^](#_ENREF_9) | +15 | +55 | 7.73 ± 1.09 | 7.14 ± 1.16 | 10.9 ± 2.46 | 8.53 ± 3.08 | 11.1 ± 1.56 | 8.06 ± 1.92 |
| Engert (2010)[^10^](#_ENREF_10) | +10 | +60 | 6.02 ± 2.23 | 3.17 ± 1.66 | 8.45 ± 3.13 | 3.17 ± 1.35 | 5.31 ± 1.89 | 2.55 ± 0.99 |
| Goldman-Melor (2012)[^12^](#_ENREF_12) | +30 | +85 | 6.33 ± 4.39 | 6.59 ± 4.49 | 7.08 ± 4.72 | 6.94 ± 4.77 | 5.35 ± 4.55 | 5.33 ± 3.78 |
| Gunnar (2009)[^14^](#_ENREF_14) | +26 | +46 | 4.41 ± 3.31 | 4.41 ± 3.31 | 6.62 ± 4.41 | 5.79 ± 4.69 | 4.41 ± 3.31 | 4.13 ± 3.03 |
| Harkness (2011)[^15^](#_ENREF_15) | +30 | +90 | 3.03 ± 1.82 | 2.78 ± 1.71 | 3.42 ± 1.94 | 3.22 ± 2.07 | 3.11 ± 1.94 | 1.89 ± 0.66 |
| McLaughlin (2015)[^18^](#_ENREF_18) | NR | NR | 6.86 ± 3.85 | 7.08 ± 3.61 | 11.30 ± 5.57 | 7.92 ± 3.90 | 8.19 ± 3.84 | 6.60 ± 2.84 |
| Mielock (2017)[^19^](#_ENREF_19) | +20 | +60 | 3.50 ± 7.75 | 1.07 ± 0.60 | 3.17 ± 5.84 | 2.48 ± 2.17 | 1.29 ± 0.66 | 1.21 ± 1.02 |
| Morris (2017)[^20^](#_ENREF_20) | +20 | +60 | 1.37 ± 1.68 | 3.42 ± 10.1 | 3.53 ± 4.11 | 2.56 ± 2.81 | 2.34 ± 4.08 | 1.40 ± 1.71 |
| Ouellet-Morin (2011)[^22^](#_ENREF_22) | +25 | +35 | 3.19 ± 0.33 | 2.9 ± 0.47 | 3.47 ± 0.33 | 3.07 ± 0.33 | 3.62 ± 0.38 | 2.88 ± 0.42 |
| Saxbe (2015)[^24^](#_ENREF_24) | +23 | +43 | 3.58 ± 3.83 | 2.97 ± 4.58 | 4.45 ± 6.19 | 3.54 ± 4.16 | 3.66 ± 5.53 | 3.17 ± 3.92 |
| Seltzer (2013)[^25^](#_ENREF_25) | +15 | +60 | 2.20 ± 0.55 | 2.06 ± 0.27 | 4.27 ± 0.96 | 1.93 ± 0.41 | 2.06 ± 0.41 | 1.51 ± 0.27 |
| Sumner (2014)[^26^](#_ENREF_26) | +30 | NR | 6.19 ± 0.93 | 7.09 ± 1.35 | 12.2 ± 1.38 | 9.74 ± 1.66 | NR | NR |
| Trickett (2014)[^27^](#_ENREF_27) | +24 | +44 | 3.31 ± 2.48 | 2.75 ± 3.03 | 4.13 ± 3.31 | 3.31 ± 3.03 | 3.31 ± 2.48 | 3.03 ± 3.31 |
| Voellmin (2015)[^28^](#_ENREF_28) | +31 | +76 | 6.07 ± 1.71 | 3.93 ± 1.74 | 12.8 ± 4.16 | 7.29 ± 3.88 | 8.49 ± 2.81 | 4.5 ± 2.63 |

*Note.* Abbreviations: NR, not reported.

**References**

1. Ali N, Pruessner JC. The salivary alpha amylase over cortisol ratio as a marker to assess dysregulations of the stress systems. *Physiol Behav* 2012; **106**(1)**:** 65-72.

2. Andreotti C, Garrard P, Venkatraman SL, Compas BE. Stress-related changes in attentional bias to social threat in young adults: Psychobiological associations with the early family environment. *Cognit Ther Res* 2015; **39**(3)**:** 332-342.

3. Armbruster D, Mueller A, Strobel A, Lesch KP, Brocke B, Kirschbaum C. Children under stress: COMT genotype and stressful life events predict cortisol increase in an acute social stress paradigm. *Int J Neuropsychopharmacol* 2012; **15**(9)**:** 1229-1239.

4. Bosch NM, Riese H, Reijneveld SA, Bakker MP, Verhulst FC, Ormel J*, et al*. Timing matters: Long term effects of adversities from prenatal period up to adolescence on adolescents' cortisol stress response. The TRAILS study. *Psychoneuroendocrinology* 2012; **37**(9)**:** 1439-1447.

5. Burkholder AR, Koss KJ, Hostinar CE, Johnson AE, Gunnar MR. Early life stress: Effects on the regulation of anxiety expression in children and adolescents. *Soc Dev* 2016; **25**(4)**:** 777-793.

6. Carnuta M, Crisan LG, Vulturar R, Opre A, Miu AC. Emotional non-acceptance links early life stress and blunted cortisol reactivity to social threat. *Psychoneuroendocrinology* 2015; **51:** 176-187.

7. Carpenter LL, Shattuck TT, Tyrka AR, Geracioti TD, Price LH. Effect of childhood physical abuse on cortisol stress response. *Psychopharmacology* 2011; **214**(1)**:** 367-375.

8. Cook EC, Chaplin TM, Sinha R, Tebes JK, Mayes LC. The stress response and adolescents' adjustment: The impact of child maltreatment. *J Youth Adolesc* 2012; **41**(8)**:** 1067-1077.

9. Elzinga BM, Roelofs K, Tollenaar MS, Bakvis P, van Pelt J, Spinhoven P. Diminished cortisol responses to psychosocial stress associated with lifetime adverse events a study among healthy young subjects. *Psychoneuroendocrinology* 2008; **33**(2)**:** 227-237.

10. Engert V, Efanov SI, Dedovic K, Duchesne A, Dagher A, Pruessner JC. Perceived early-life maternal care and the cortisol response to repeated psychosocial stress. *J Psychiatry Neurosci* 2010; **35**(6)**:** 370-377.

11. Fan Y, Pestke K, Feeser M, Aust S, Pruessner JC, Boker H*, et al*. Amygdala-hippocampal connectivity changes during acute psychosocial stress: Joint effect of early life stress and oxytocin. *Neuropsychopharmacology* 2015; **40**(12)**:** 2736-2744.

12. Goldman-Mellor S, Hamer M, Steptoe A. Early-life stress and recurrent psychological distress over the lifecourse predict divergent cortisol reactivity patterns in adulthood. *Psychoneuroendocrinology* 2012; **37**(11)**:** 1755-1768.

13. Gordis EB, Granger DA, Susman EJ, Trickett PK. Salivary alpha amylase-cortisol asymmetry in maltreated youth. *Horm Behav* 2008; **53**(1)**:** 96-103.

14. Gunnar MR, Frenn K, Wewerka SS, Van Ryzin MJ. Moderate versus severe early life stress: Associations with stress reactivity and regulation in 10-12-year-old children. *Psychoneuroendocrinology* 2009; **34**(1)**:** 62-75.

15. Harkness KL, Stewart JG, Wynne-Edwards KE. Cortisol reactivity to social stress in adolescents: Role of depression severity and child maltreatment. *Psychoneuroendocrinology* 2011; **36**(2)**:** 173-181.

16. Houtepen LC, Vinkers CH, Carrillo-Roa T, Hiemstra M, van Lier PA, Meeus W*, et al*. Genome-wide DNA methylation levels and altered cortisol stress reactivity following childhood trauma in humans. *Nat Commun* 2016; **7:** 10967.

17. MacMillan HL, Georgiades K, Duku EK, Shea A, Steiner M, Niec A*, et al*. Cortisol response to stress in female youths exposed to childhood maltreatment: Results of the Youth Mood Project. *Biol Psychiatry* 2009; **66**(1)**:** 62-68.

18. McLaughlin KA, Sheridan MA, Tibu F, Fox NA, Zeanah CH, Nelson CA, 3rd. Causal effects of the early caregiving environment on development of stress response systems in children. *Proc Natl Acad Sci U S A* 2015; **112**(18)**:** 5637-5642.

19. Mielock AS, Morris MC, Rao U. Patterns of cortisol and alpha-amylase reactivity to psychosocial stress in maltreated women. *J Affect Disord* 2017; **209:** 46-52.

20. Morris MC, Kouros CD, Mielock AS, Rao U. Depressive symptom composites associated with cortisol stress reactivity in adolescents. *J Affect Disord* 2017; **210:** 181-188.

21. Mueller A, Armbruster D, Moser DA, Canli T, Lesch KP, Brocke B*, et al*. Interaction of serotonin transporter gene-linked polymorphic region and stressful life events predicts cortisol stress response. *Neuropsychopharmacology* 2011; **36**(7)**:** 1332-1339.

22. Ouellet-Morin I, Odgers CL, Danese A, Bowes L, Shakoor S, Papadopoulos AS*, et al*. Blunted cortisol responses to stress signal social and behavioral problems among maltreated/bullied 12-year-old children. *Biol Psychiatry* 2011; **70**(11)**:** 1016-1023.

23. Peckins MK, Dockray S, Eckenrode JL, Heaton J, Susman EJ. The longitudinal impact of exposure to violence on cortisol reactivity in adolescents. *J Adolesc Health* 2012; **51**(4)**:** 366-372.

24. Saxbe DE, Negriff S, Susman EJ, Trickett PK. Attenuated hypothalamic-pituitary-adrenal axis functioning predicts accelerated pubertal development in girls 1 year later. *Dev Psychopathol* 2015; **27**(3)**:** 819-828.

25. Seltzer LJ, Ziegler T, Connolly MJ, Prososki AR, Pollak SD. Stress-induced elevation of oxytocin in maltreated children: Evolution, neurodevelopment, and social behavior. *Child Dev* 2014; **85**(2)**:** 501-512.

26. Sumner JA, McLaughlin KA, Walsh K, Sheridan MA, Koenen KC. CRHR1 genotype and history of maltreatment predict cortisol reactivity to stress in adolescents. *Psychoneuroendocrinology* 2014; **43:** 71-80.

27. Trickett PK, Gordis E, Peckins MK, Susman EJ. Stress reactivity in maltreated and comparison male and female young adolescents. *Child Maltreat* 2014; **19**(1)**:** 27-37.

28. Voellmin A, Winzeler K, Hug E, Wilhelm FH, Schaefer V, Gaab J*, et al*. Blunted endocrine and cardiovascular reactivity in young healthy women reporting a history of childhood adversity. *Psychoneuroendocrinology* 2015; **51:** 58-67.

29. Wingenfeld K, Kuehl LK, Boeker A, Schultebraucks K, Ritter K, Hellmann-Regen J*, et al*. Stress reactivity and its effects on subsequent food intake in depressed and healthy women with and without adverse childhood experiences. *Psychoneuroendocrinology* 2017; **80:** 122-130.

30. Hostinar CE, Johnson AE, Gunnar MR. Early social deprivation and the social buffering of cortisol stress responses in late childhood: An experimental study. *Dev Psychol* 2015; **51**(11)**:** 1597-1608.
